# Neurologic manifestations of COVID-19 in critically ill patients: results of the prospective multicenter registry PANDEMIC

**DOI:** 10.1186/s13054-022-04080-3

**Published:** 2022-07-16

**Authors:** Konstantinos Dimitriadis, Jan Meis, Hermann Neugebauer, Kristian Barlinn, Bernhard Neumann, Georg Gahn, Piergiorgio Lochner, Benjamin Knier, Sarah Lindemann, Kurt Wolfram Sühs, Kristina Szabo, Thomas Pfefferkorn, Ingo Schirotzek, Tobias Freilinger, Bassa Burc, Albrecht Günther, Matthias Wittstock, Patrick Schramm, Gernot Reimann, Jana Godau, Gabor Nagy, Fatima B. Koenig, Fabian Essig, Hartwig Klinker, Christian Hartmann, Moritz L. Schmidbauer, Tim Steinberg, Lora Lefterova, Christina Klose, Julian Bösel, for the IGNITE study group

**Affiliations:** 1 Department of Neurology, University Hospital LMU Munich, Munich, Germany; 2 Institute for Stroke and Dementia Research (ISD), LMU Munich, Munich, Germany; 3 Institute of Medical Biometry, University of Heidelberg, Heidelberg, Germany; 4 Department of Neurology, University Hospital Würzburg, Würzburg, Germany; 5 Department of Neurology, University Hospital Carl Gustav Carus, Technische Universität Dresden, Dresden, Germany; 6 Present Address: Department of Neurology, Donau-Isar-Klinikum Deggendorf, Deggendorf, Germany; 7 Department of Neurology, University of Regensburg, Bezirksklinikum Regensburg, Regensburg, Germany; 8 Department of Neurology, Klinikum Karlsruhe, Karlsruhe, Germany; 9 Department of Neurology, Saarland University Medical Center, Homburg, Germany; 10 Department of Neurology, Klinikum rechts der Isar, Technical University of Munich, Munich, Germany; 11 SRH-Waldklinikum Gera, Gera, Germany; 12 Department of Neurology, Medical School Hannover, Hannover, Germany; 13 Department of Neurology, Medical Faculty Mannheim and Mannheim Center for Translational Neurosciences (MCTN), Heidelberg University, Mannheim, Germany; 14 Department of Neurology, Klinikum Ingolstadt, Ingolstadt, Germany; 15 Department of Neurology, Klinikum Darmstadt, Darmstadt, Germany; 16 Klinik für Neurologie, Klinikum Passau, Passau, Germany; 17 Department of Neurology, Krankenhaus Nordwest, Frankfurt, Germany; 18 Department of Neurology, Jena University Hospital, Jena, Germany; 19 Department of Neurology, University Medicine Rostock, Rostock, Germany; 20 Department of Neurology, Universitätätsklinikum Giessen und Marburg, Standort Giessen, Justus-Liebig-University, Giessen, Germany; 21 Department of Neurology, Klinikum Dortmund gGmbH, Dortmund, Germany; 22 Department of Neurology, Klinikum Kassel, Kassel, Germany; 23 Department of Internal Medicine II, Division of Infectious Diseases, University Hospital Würzburg, Würzburg, Germany; 24 Department of Neurology, Heidelberg University Hospital, Heidelberg, Germany

**Keywords:** COVID-19, Neurologic manifestations, Intensive care, Critically ill

## Abstract

**Background:**

Neurologic manifestations are increasingly reported in patients with coronavirus disease 2019 (COVID-19). Yet, data on prevalence, predictors and relevance for outcome of neurological manifestations in patients requiring intensive care are scarce. We aimed to characterize prevalence, risk factors and impact on outcome of neurologic manifestations in critically ill COVID-19 patients.

**Methods:**

In the prospective, multicenter, observational registry study PANDEMIC (Pooled Analysis of Neurologic DisordErs Manifesting in Intensive care of COVID-19), we enrolled COVID-19 patients with neurologic manifestations admitted to 19 German intensive care units (ICU) between April 2020 and September 2021. We performed descriptive and explorative statistical analyses. Multivariable models were used to investigate factors associated with disorder categories and their underlying diagnoses as well as to identify predictors of outcome.

**Results:**

Of the 392 patients included in the analysis, 70.7% (277/392) were male and the mean age was 65.3 (SD ± 3.1) years. During the study period, a total of 2681 patients with COVID-19 were treated at the ICUs of 15 participating centers. New neurologic disorders were identified in 350 patients, reported by these centers, suggesting a prevalence of COVID-19-associated neurologic disorders of 12.7% among COVID-19 ICU patients. Encephalopathy (46.2%; 181/392), cerebrovascular (41.0%; 161/392) and neuromuscular disorders (20.4%; 80/392) were the most frequent categories identified. Out of 35 cerebrospinal fluid analyses with reverse transcriptase PCR for SARS-COV-2, only 3 were positive. In-hospital mortality was 36.0% (140/389), and functional outcome (mRS 3 to 5) of surviving patients was poor at hospital discharge in 70.9% (161/227). Intracerebral hemorrhage (OR 6.2, 95% CI 2.5–14.9, *p* < 0.001) and acute ischemic stroke (OR 3.9, 95% CI 1.9–8.2, *p* < 0.001) were the strongest predictors of poor outcome among the included patients.

**Conclusions:**

Based on this well-characterized COVID-19 ICU cohort, that comprised 12.7% of all severe ill COVID-19 patients, neurologic manifestations increase mortality and morbidity. Since no reliable evidence of direct viral affection of the nervous system by COVID-19 could be found, these neurologic manifestations may for a great part be indirect para- or postinfectious sequelae of the infection or severe critical illness. Neurologic ICU complications should be actively searched for and treated.

**Supplementary Information:**

The online version contains supplementary material available at 10.1186/s13054-022-04080-3.

## Introduction

Infection with severe acute respiratory syndrome coronavirus 2 (SARS-CoV-2) causing the coronavirus disease 2019 (COVID-19) renders 10–15% of COVID-19 patients to require intensive care unit (ICU) treatment [1–3]. Although respiratory failure and multi-organ dysfunction are the most common indications for ICU admission, about 1/3 of all COVID-19 patients are reported to present with neurologic manifestations [3–9]. Furthermore, neurologic manifestations have been suggested as predictors of mortality and functional outcome [9–12].

Several pathophysiological mechanisms have been proposed to explain neurological manifestations in COVID-19 [5, 13–15]. However, as ICU patients not suffering from COVID-19 also frequently exhibit neurologic complications like encephalopathy, critical illness polyneuropathy/myopathy (CIP/CIM) or cerebrovascular disorders, the evaluation of a direct pathophysiological link between the virus and neurologic complications is challenging [16–19]. Furthermore, studies on neurologic manifestations of COVID-19 in- and outside the ICU are largely hampered by their retrospective design and its inherent biases, discrepancies in methodology, differences in definitions of neurologic disorders, sample size and reporting bias. As a result, data on rates, prevalence and relevance vary greatly and leave uncertainty, particularly regarding the ICU population.

We aimed to characterize critically ill COVID-19 patients in a prospective, multicenter, observational cohort study employing neurology consultations. Our aims were first, to assess the prevalence of neurologic manifestations, second, to systematically evaluate clinical characteristics, third, to identify predictors of neurological disease, and fourth, to evaluate the prognostic relevance of neurologic manifestations to overall mortality and functional outcome.

## Methods

### PANDEMIC registry and study design

PANDEMIC (Pooled Analysis of Neurologic DisordErs Manifesting in Intensive care of COVID-19) is a registry study conducted by the research network IGNITE (Initiative of German NeuroIntensive Trial Engagement) with support of the German Society for Neurologic Intensive Care and Emergency Medicine (DGNI). Local ethics committees and institutional review boards of the participating centers approved the study based on the central vote of the ethics committee of Landesärztekammer Hessen, Germany (state medical association, 2020-1619-evBO, ethikkommission@laekh.de).

### Setting

Patients with SARS-CoV-2 infection confirmed by polymerase chain reaction (PCR) were admitted to ICUs of participating centers (mostly general ICUs) and treated at the discretion of local physicians. Neurologists with experience in neurocritical care were consulted when neurologic manifestations occurred. Diagnostic investigations were ordered either before or because of those consultations. A list of all recorded parameters is provided in the supplement.

### Patients

The trial was active between April 2020 and September 2021. Inclusion criteria were age > 18 years, ICU admission, confirmed SARS-CoV-2 infection and at least one new neurologic or psychiatric manifestation that triggered a neurology consultation. Patients with preexisting neurologic disease and without new symptoms were excluded.

### Categories of disorders and underlying diagnoses

Utilizing an inductive categorization process using all available data, we mapped the diverse set of neurologic manifestations (with the term “neurologic manifestations” we summarized signs, symptoms and diagnoses) observed in our cohort to the following categories of disease: 1. cerebrovascular disorder (CV), 2. neuromuscular disorder (NMD), 3. encephalopathy, 4. inflammatory central nervous system disorder (CNS), 5. epileptic disorder, and 6. others. A detailed list of subcategories and definitions is provided in Table 1 and the supplement.

**Table 1:** Baseline characteristics according to disease categories in 392 patients

Disease categories^b^							
	CV disorders	Neuromuscular disorder	Encephalopathy	Inflamatory CNS disorders	Epileptic disorders	Other	Total
N^a^	161	80	181	7	40	61	392
Demographics							
Age (mean ± SD)	67.0 ± 13.4	61.6 ± 12.8	66.6 ± 12.7	61.9 ± 9.3	62.5 ± 13.3	60.3 ± 9.7	65.3 ± 13.1
Sex							
Male	112 (69.6%)	59 (73.8%)	131 (72.4%)	6 (85.7%)	27 (67.5%)	43 (70.5%)	277 (70.7%)
Female	49 (30.4%)	21 (26.3%)	50 (27.6%)	1 (14.3%)	13 (32.5%)	18 (29.5%)	115 (29.3%)
Nicotine consumption (*N* = 184)	20 (25.3%)	12 (28.6%)	22 (31.0%)	0 (0.0%)	8 (36.4%)	10 (28.6%)	50 (27.2%)
Alcohol consumption^c^ (*N* = 260)	8 (7.3%)	2 (3.8%)	10 (8.5%)	0 (0.0%)	5 (14.7%)	1 (2.2%)	17 (6.5%)
Past medical history							
Neurological disease^d^ (*N* = 386)	47 (29.7%)	14 (17.5%)	43 (24.3%)	2 (28.6%)	11 (28.2%)	11 (18.3%)	88 (22.8%)
Cardiovascular disease (*N* = 375)	105 (68.2%)	48 (61.5%)	124 (72.5%)	3 (42.9%)	25 (65.8%)	36 (63.2%)	262 (69.9%)
Coronary heart disease (*N* = 383)	30 (19.5%)	6 (7.9%)	28 (16.5%)	0 (0.0%)	2 (5.4%)	6 (10.5%)	54 (14.5%)
Lung disease (*N* = 368)	28 (18.5%)	8 (10.8%)	38 (22.5%)	2 (28.6%)	7 (18.4%)	8 (14.0%)	69 (18.8%)
Diabetes (*N* = 373)	47 (30.5%)	20 (26.3%)	46 (27.1%)	0 (0.0%)	12 (32.4%)	19 (33.3%)	110 (29.5%)
Dyslipidemia (*N* = 373)	30 (19.5%)	11 (14.5%)	29 (17.1%)	0 (0.0%)	7 (18.9%)	9 (15.8%)	63 (16.9%)
Hypertension (*N* = 373)	87 (56.5%)	38 (50.0%)	104 (61.2%)	2 (33.3%)	20 (54.1%)	30 (52.6%)	224 (60.1%)
Pre-hospital medications							
Anticoagulant drugs (*N* = 322)	50 (36.2%)	15 (22.7%)	53 (35.8%)	1 (14.3%)	12 (38.7%)	14 (26.9%)	107 (33.2%)
Immunosuppressants (*N* = 376)	18 (11.8%)	8 (10.1%)	21 (12.2%)	1 (14.3%)	9 (23.1%)	9 (15.3%)	45 (12.0%)
pmRS (*N* = 322)							
0	90 (65.2%)	38 (57.6%)	79 (52.3%)	3 (42.9%)	17 (50.0%)	42 (84.0%)	197 (61.2%)
1	16 (11.6%)	13 (19.7%)	32 (21.2%)	2 (28.6%)	8 (23.5%)	5 (10.0%)	49 (15.2%)
2	8 (5.8%)	6 (9.1%)	15 (9.9%)	1 (14.3%)	3 (8.8%)	1 (2.0%)	25 (7.8%)
3	6 (4.3%)	2 (3.0%)	11 (7.3%)	0 (0.0%)	3 (8.8%)	1 (2.0%)	20 (6.2%)
4	8 (5.8%)	4 (6.1%)	11 (7.3%)	0 (0.0%)	1 (2.9%)	0 (0.0%)	18 (5.6%)
5	10 (7.2%)	3 (4.5%)	3 (2.0%)	1 (14.3%)	2 (5.9%)	1 (2.0%)	13 (4.0%)

pmRS: premorbid modified Ranking Score (pmRS score; a 7-point scale reflecting daily functioning ranging from 0 (full independence without symptoms) to 6 (death)). SD: Standard Deviation

^a^Number of data sets used for analysis is mentioned, where N < 392 (total number of patients included), values are missing

^b^Cerebrovascular disorder (= ischemic stroke, intracerebral hemorrhage, subarachnoid hemorrhage, posterior reversible encephalopathy syndrome, reversible cerebral vasoconstriction syndrome, cerebral venous sinus thrombosis, cerebral microbleeds, subdural hematoma); neuromuscular disorder (NMD)(= critical illness polyneuropathy or myopathy, Guillain–Barré syndrome, myasthenia, myositis); encephalopathy (= delirium, disorder of consciousness, hypoxic encephalopathy, encephalopathy no further described); inflammatory CNS disorder (= meningitis, encephalitis, meningoencephalitis, herpes zoster oticus, polyneuritis cranialis); epileptic disorder (= seizures, status epilepticus); other (= brain edema, exophthalmus, tetraparesis, facial palsy, plexus lesion, phobic gait disorder, major depression, restless legs syndrome, gaze palsy, fine motor impairment)

^c^More than two standard drinks a day

^d^Neurologic disorder included (main categories: ischemic stroke, hemorrhagic stroke, subarachnoid hemorrhage, dementia, epileptic disorder, Parkinson’s disease, traumatic brain injury, myasthenia gravis, multiple sclerosis, others)

### Definition of outcomes

Next to mortality, functional outcome was measured by the modified Rankin Scale (mRS). mRS of 0–2 was defined as good functional status.

### Statistical analyses

Parameters were recorded in an electronic case report form (eCRF). The data were then reviewed for internal validity, and records with insufficient documentation were removed.

Descriptive statistics were calculated for each of the disease categories, as well as for the most common diagnoses, respectively. The categories were allowed to have an overlap of data between each other, as patients may exhibit symptoms of different disease categories. The prevalence of neurologic manifestations was estimated as the fraction of patients with neurologic manifestations in the population of ICU COVID-19 patients from data of 15 (which could provide data on total number of COVID-19 ICU admissions) out of 19 centers. Potential explanatory variables for mortality were identified by literature review and clinical reasoning (Table 4 and supplement). These variables were evaluated together with the categories of disease in a multivariable logistic regression model using a multiple imputation approach according to the fully conditional specification method to handle missing data. Furthermore, the association of various factors with the most common diagnoses was analyzed using a similar model to the one described above. Analyses were conducted following an exploratory strategy, and all p values are reported as descriptive measures without adjustment for multiple testing. Analyses were performed using SAS version 9.4 (SAS Institute) and R^54^ version 4.0.3.

## Results

### Baseline characteristics

**Fig. 1:**
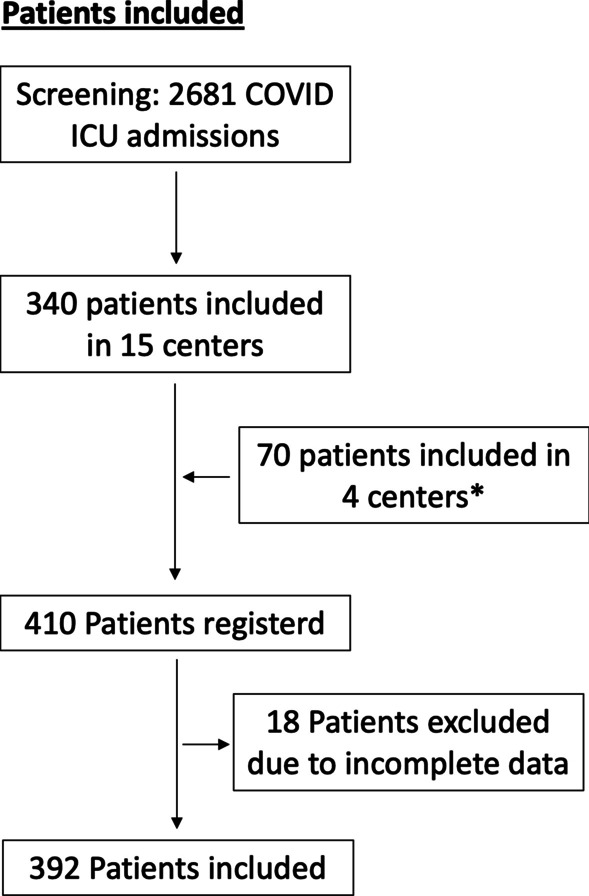
Patients included. *Number of total ICU admission in 4 centers missing

Of all centers, 15 reported 2,681 SARS-CoV-2 patients admitted to their ICUs during the study period (number of total ICU admission in 4 centers missing), out of which 340 developed neurologic manifestations, yielding a prevalence of 12.7%. The remaining 4 centers included 70 ICU patients with neurologic manifestations. (In those centers, no information on number of total ICU admission was available.) Therefore, a total of 410 COVID-19 ICU patients were registered by all 19 German centers. All had a positive PCR result with a median of 10 days from first positive testing (IQR 0–24) to the onset of neurologic manifestations. Due to insufficient documentation, 18 patients were excluded (Fig. 1).

Demographic and clinical characteristics of patients are presented in Table 1. Of all analyzed patients, 70.7% (277/392) were male and mean age was 65.3 (standard deviation SD ± 13.1) years. Eighty-four percent of the patients had a good functional status prior to ICU admission (61.2%; 197/322 mRS 0, 15.2%; 49/322 mRS 1 and 7.8%; 25/322 mRS 2) (Table 1).

Only 22.8% (88/386) of patients had a known preexisting neurological disease, with cerebrovascular disorders being most prevalent (9.6%; 37/386) (Additional file 1: Table S1). The most common reason for inclusion in the study was a new onset neurologic deficit (90.4%; 339/375). Moreover, 17.6% of patients were included with new psychiatric symptoms (17.6%; 61/347) (Additional file 1: Table S2).

### ICU characteristics

Indications for ICU admission, complications during the ICU treatment as well as diseases’ severity scores are displayed in Table 2. The most common indication was respiratory failure (80.6%; 312/387). Furthermore, 17.5% (68/387) were admitted due to a new neurologic deficit, most frequently associated with a cerebrovascular disorder (70%; 48/69) (Table 2).The Lean European Open Survey for SARS-CoV-2 Infected Patients (LEOSS) stage of disease on ICU admission was classified as critical for almost half of the patients (48.5%, 141/291) (Additional file 1: Table S2) [7]. Moreover, half of the patients admitted due to neurologic deficits (34/68) were classified as critical or complicated according to the LEOSS stage of disease algorithm. Data on use of invasive ventilation and vasopressors, as well as rates of organ failure and complications, are presented in Table 2.

**Table 2:** ICU admission and ICU course of disease according to disease categories

	CV disorder	Neuromuscular disorder	Encephalopathy	Infl. CNS disorder	Epileptic disorder	Other	Total
N	161	80	181	7	40	61	392
Reason for admission (*N* = 387)							
Respiratory failure	106 (66.7%)	71 (91.0%)	153 (85.5%)	5 (71.4%)	34 (87.2%)	38 (88.4%)	312 (80.6%)
Circulatory failure	18 (11.3%)	11 (14.1%)	23 (12.8%)	1 (14.3%)	2 (5.1%)	6 (14.0%)	40 (10.3%)
Impaired consciousness	14 (8.8%)	1 (1.3%)	19 (10.6%)	1 (14.3%)	4 (10.3%)	3 (7.0%)	33 (8.5%)
Other reasons	48 (30.2%)	9 (11.5%)	19 (10.6%)	2 (28.6%)	7 (17.9%)	3 (7.0%)	69 (17.8%)
ICU course of disease							
Vasopressors (*N* = 388)	126 (78.8%)	72 (90.0%)	146 (82.0%)	6 (85.7%)	30 (76.9%)	41 (97.6%)	315 (81.2%)
Invasive ventilation (*N* = 389)	131 (81.9%)	75 (93.8%)	142 (79.3%)	6 (85.7%)	30 (75.0%)	41 (97.6%)	319 (82.0%)
ARDS (*N* = 388)	98 (61.3%)	69 (87.3%)	135 (75.8%)	6 (85.7%)	26 (65.0%)	36 (85.7%)	277 (71.4%)
Use of ECMO (*N* = 385)	47 (29.6%)	28 (35.4%)	31 (17.6%)	2 (28.6%)	9 (22.5%)	20 (47.6%)	91 (23.6%)
Sepsis (*N* = 385)	81 (50.6%)	52 (65.8%)	103 (58.5%)	3 (42.9%)	24 (61.5%)	28 (66.7%)	212 (55.1%)
Acute kidney failure (*N* = 386)	67 (41.9%)	39 (49.4%)	88 (49.7%)	5 (71.4%)	20 (51.3%)	26 (61.9%)	172 (44.6%)
Acute liver failure^a^ (*N* = 378)	27 (17.3%)	8 (10.5%)	22 (12.6%)	0 (0.0%)	4 (10.5%)	6 (14.3%)	48 (12.7%)
Scores (mean ± SD)							
GCS (Min.) (*N* = 350)	7.5 ± 4.6	11.3 ± 4.3	10.1 ± 4.2	10.0 ± 4.2	8.1 ± 4.8	7.7 ± 5.2	9.8 ± 4.7
RASS (Min.) (*N* = 343)	− 2.6 ± 2.2	− 1.0 ± 1.7	− 1.3 ± 2.3	− 0.8 ± 1.3	− 2.6 ± 2.3	− 2.7 ± 2.3	− 1.6 ± 2.3
RASS (Max.) (*N* = 343)	− 2.3 ± 2.3	− 0.7 ± 1.5	− 0.9 ± 2.2	− 0.6 ± 1.1	− 2.0 ± 2.3	− 2.6 ± 2.3	− 1.3 ± 2.2
FOUR (Min.) (*N* = 254)	8.3 ± 5.8	12.2 ± 3.7	11.3 ± 4.7	11.2 ± 3.8	8.9 ± 5.7	7.1 ± 6.7	10.8 ± 5.3
SAPS (Max.) (*N* = 155)	49.5 ± 20.5	38.4 ± 17.4	45.1 ± 16.8	39.0 ± 18.4	41.6 ± 17.3	46.2 ± 18.4	43.6 ± 18.0
SOFA respiratory system (Max.) (*N* = 187)	2.5 ± 1.2	2.2 ± 0.9	2.4 ± 1.0	1.8 ± 1.0	2.6 ± 1.3	2.8 ± 1.2	2.4 ± 1.1
SOFA circulatory system (Max.) (*N* = 178)	2.3 ± 1.4	1.7 ± 1.1	2.1 ± 1.2	1.0 ± 0.0	2.6 ± 1.4	2.5 ± 1.3	2.2 ± 1.3
Time from onset of COVID symptoms to first neurologic symptoms (Days; Median (IQR)) (*N* = 322)	10 (1, 23)	26 (20, 38)	12 (3, 25)	30 (25, 41)	8 (4, 25)	13.5 (0.5, 26)	13 (3, 25)

ECMO: extracorporeal membrane oxygenation, ARDS: acute respiratory distress syndrome, SOFA: Sequential Organ Failure Assessment Score, SAPS: Simplified Acute Physiology Score, RASS: Richmond Agitation-Sedation Scale, GCS: Glasgow Coma Scale, and FOUR: Full Outline of UnResponsiveness

^a^(Quick < 50%, ALT/AST > 5 × ULN)

### Neurologic symptoms

New neurologic symptoms occurred in 90.4% (339/375) of the patients and are summarized in Additional file 1: Table S3. The most common neurologic symptoms were impaired consciousness (58.2%; 228/392) and new motor deficits (47.7%; 187/392). On average, neurologic symptoms were detected after a median of 13 days (interquartile range IQR 3–25) after the first COVID-19 symptom. Symptoms associated with neuromuscular disorders or inflammatory disorders appeared later with a median of 26 (IQR 20–38) and 30 (IQR 25–41) days, respectively.

### Diagnostic findings

**Fig. 2:**
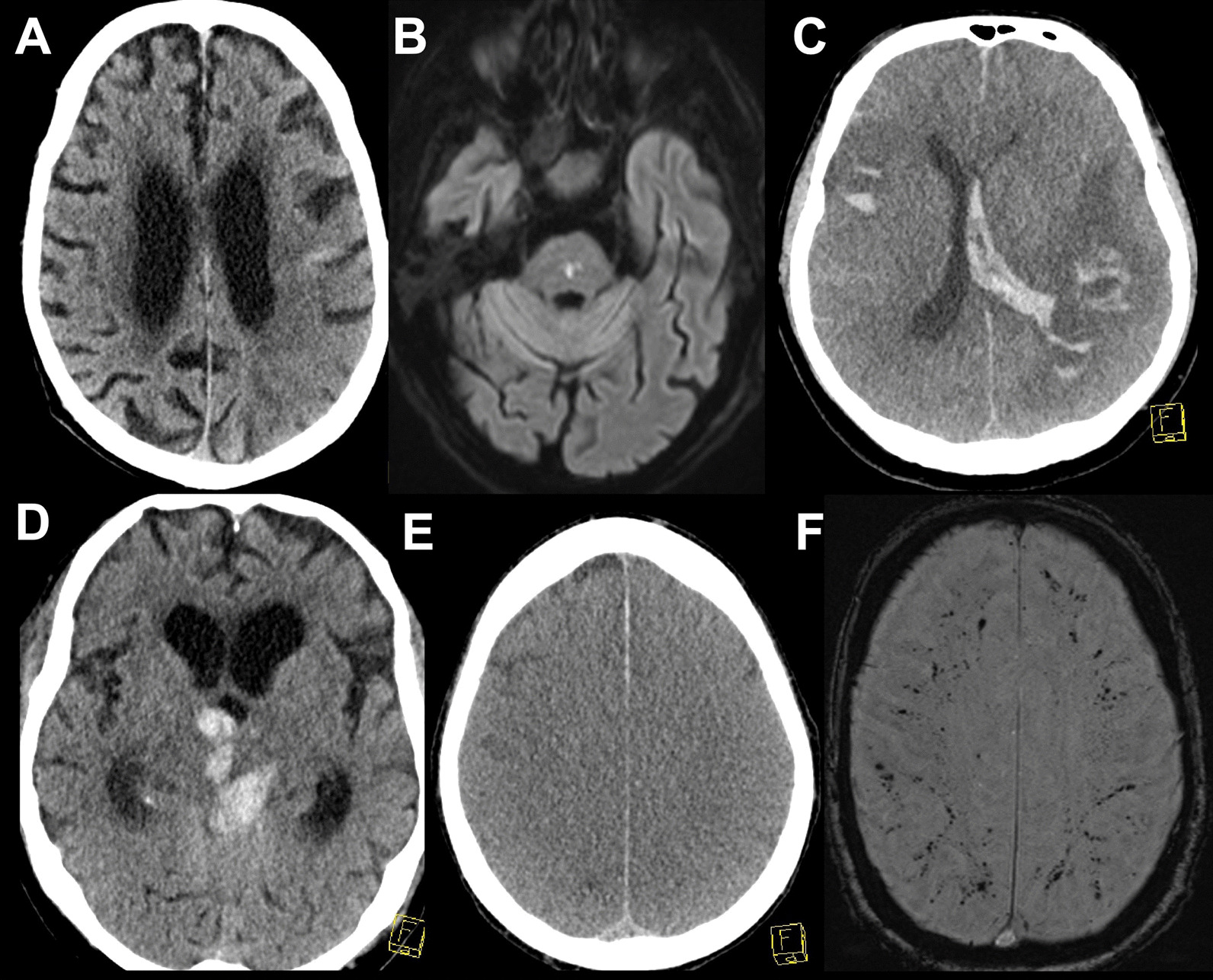
Characteristic imaging findings. **A** Computed tomography scan (CT) of a 90-year-old male patient with left parietal infarction. **B** Diffusion-weighted magnetic resonance imaging (DWI–MRI) of an 86-year-old male patient with a pontine infarction. **C** CT scan of a 67-year-old male patient with multiple intracerebral hemorrhages and intraventricular extension. **D** CT scan of a 78-year-old male patient with intracerebral hemorrhage with intraventricular extension. **E** CT scan of a 48-year-old female patient with severe hypoxia and generalized edema. **F** Susceptibility-weighted angiography MRI images of a 66-year-old male patient with multiple microbleeds

Lumbar puncture was performed in 44 patients, and cerebrospinal fluid (CSF) analysis yielded a slight-to-moderate pleocytosis (7–50 cells/µl) in 4 patients, severe pleocytosis in 1 patient (2,734 cells/µl) and normal cell counts in the rest. A SARS-COV-2 PCR was performed in 35 CSF specimens and proved positive in three patients, of which only 1 revealed abnormal cell counts with 17 cells/µl. Neuroradiologic imaging comprised cranial computer tomography (CCT) in 264 (71 patients had at least 2 CCTs during their ICU stay) and magnetic resonance imaging (MRI) in 71 of the patients and was pathologic in 158/264 (59.8%) and 55/71 (77.5%), respectively (characteristic examples are illustrated in Fig. 2, Additional file 1: Table S4). Electrophysiologic investigations were performed in 95 patients (electromyography in 21 patients, evoked potentials in 7 patients, electroencephalography (EEG) in 62 patients), which revealed pathological findings in > 90.1% of cases (95.2% of electromyography findings, 100% of evoked potential findings and 81.8% of EEG findings). The most common EEG finding was encephalopathy in 61.3% of patients.

### Neurologic manifestations

Among neurologic manifestations, encephalopathy (46.2%; 181/392), CV (41.1%; 161/392) and NMD (20.0%; 80/392) as superordinate categories were most frequently observed (Table 1). The most common neurological diagnoses underlying the categories were delirium (29.6%; 116/392), acute ischemic stroke (AIS) (27.3%; 107/392 and prevalence of 3.5%; 93/2,681), CIP or CIM (17.6%; 69/392) and intracerebral hemorrhage (ICH) (13.8%; 54/392 and prevalence of 1.8%; 49/2681). Moreover, subarachnoid hemorrhage (SAH) occurred with a prevalence of 1.2% (31/2681) and the combined prevalence of ICH and SAH was 3.0% (80/2681).

### Predictors of neurological manifestations

In a multivariable regression model (*n* = 392), use of extracorporeal membrane oxygenation (ECMO), invasive ventilation, acute liver failure and older age were independently associated with CV (Additional file 1: Table S5). Moreover, and for the occurrence of AIS, older age and a history of ischemic stroke were independent predictors. Known cardiovascular risk factors like hypertension, diabetes mellitus, nicotine consumption or dyslipidemia did not have a significant influence (Additional file 1: Table S6a). Occurrence of ICH was associated with ECMO therapy and elevated activated partial thromboplastin time (aPTT) measures (Additional file 1: Table S6b).

Apart from the time to first neurologic deficit since COVID-19 diagnosis, no other factors predicting the category of NMD could be identified (Additional file 1: Table S5). However, ARDS significantly increased the risk of CIP/CIM (Additional file 1: Table S6).

Furthermore, ARDS independently predicted the category encephalopathy (Additional file 1: Table S5). For the underlying diagnosis of delirium, older age was a predictor, while female sex was associated with a lower risk (Additional file 1: Table S6).

### Outcomes

The median hospital and ICU length of stay were 28 (IQR 15–44) and 20 (IQR 10–35) days, respectively. 36.0% (140/389) of the patients died (25.4%; 96/378 received palliative care, but not all had died at the end of observation). The decision to limit therapy, analyzed in a multivariable model (*n* = 392), was only influenced by older age and new cerebrovascular disorder (Additional file 1: Table S7). For 39 (27.9%) of the 140 deceased patients, a neurologic cause of death was documented. Most survivors were discharged to a rehabilitation facility (28.5%; 111/389), while only 17.7% (69/389) were discharged home. Most of the survivors did not reach functional independence on discharge (Table 3).

**Table 3:** Outcomes

	CV disorders	Neuromuscular disorder	Encephalopathy	Infl. CNS disorders	Epileptic disorders	Other	Total
N	161	80	181	7	40	61	392
Length of stay (days; median (IQR))							
Hospital (*N* = 369)	22 (10, 41)	37 (28, 56)	29.5 (17, 47)	37 (32, 51)	38 (24, 47)	26 (13, 44)	28 (15, 44)
ICU (*N* = 364)	14 (5, 33)	29 (19.5, 44)	20 (11, 40)	36 (29, 51)	19.5 (14, 38)	22 (12, 38)	20 (9.5, 35)
Discharge mode (*N* = 389)							
Home care	15 (9.3%)	10 (12.8%)	33 (18.2%)	0 (0.0%)	8 (20.5%)	14 (23.0%)	69 (17.7%)
Transfer to another hospital	12 (7.5%)	20 (25.6%)	36 (19.9%)	2 (33.3%)	4 (10.3%)	3 (4.9%)	61 (15.7%)
Rehabilitation center	36 (22.4%)	39 (50.0%)	47 (26.0%)	3 (50.0%)	10 (25.6%)	19 (31.1%)	111 (28.5%)
Nursing home	3 (1.9%)	0 (0.0%)	5 (2.8%)	0 (0.0%)	3 (7.7%)	0 (0.0%)	8 (2.1%)
Death	95 (59.0%)	9 (11.5%)	60 (33.1%)	1 (16.7%)	14 (35.9%)	25 (41.0%)	140 (36.0%)
mRS on discharge (v367)							
0	3 (1.9%)	0 (0.0%)	5 (2.9%)	0 (0.0%)	2 (5.6%)	4 (6.9%)	13 (3.5%)
1	9 (5.8%)	2 (2.7%)	15 (8.8%)	0 (0.0%)	5 (13.9%)	5 (8.6%)	29 (7.9%)
2	7 (4.5%)	2 (2.7%)	14 (8.2%)	0 (0.0%)	4 (11.1%)	3 (5.2%)	24 (6.5%)
3	5 (3.2%)	10 (13.7%)	12 (7.1%)	1 (14.3%)	3 (8.3%)	6 (10.3%)	28 (7.6%)
4	17 (11.0%)	21 (28.8%)	34 (20.0%)	3 (42.9%)	6 (16.7%)	6 (10.3%)	65 (17.7%)
5	19 (12.3%)	29 (39.7%)	30 (17.6%)	2 (28.6%)	2 (5.6%)	9 (15.5%)	68 (18.5%)
6	95 (61.3%)	9 (12.3%)	60 (35.3%)	1 (14.3%)	14 (38.9%)	25 (43.1%)	140 (38.1%)
Cause of death (*N* = 139)							
Neurological cause	37 (39.4%)	1 (11.1%)	9 (15.0%)	0 (0.0%)	3 (21.4%)	17 (68.0%)	39 (28.1%)
Respiratory failure	20 (21.3%)	3 (33.3%)	21 (35.0%)	1 (100.0%)	6 (42.9%)	4 (16.0%)	41 (29.5%)
Other reasons	32 (34.0%)	4 (44.4%)	25 (41.7%)	0 (0.0%)	5 (35.7%)	4 (16.0%)	51 (36.7%)
Unclear	5 (5.3%)	1 (11.1%)	5 (8.3%)	0 (0.0%)	0 (0.0%)	0 (0.0%)	8 (5.8%)
Initiation of palliative care (*N* = 378)	62 (40.3%)	6 (7.8%)	43 (24.6%)	1 (14.3%)	10 (25.0%)	18 (30.5%)	96 (25.4%)

ICU: Intensive care unit, mRS: modified ranking scale

The strongest predictor of death was the occurrence of CV (OR 8.2, 95% CI 3.8–17.3) followed by the use of vasopressors (OR 6.9, 95% CI 1.8–26.4) and acute liver failure (OR 4.2, 95% CI 1.6–11.1). Other factors are displayed in Table 4 (Table 4). With regards to specific diseases, ICH was associated with the highest risk of death (OR 6.1, 95% CI 2.5–14.9), followed by AIS (OR 3.9, 95% CI 1.9–8.2). Both prevailed when adjusting for the confounders age, liver failure, ECMO, higher pmRS and vasopressors, which remained significant in their prediction of mortality (Table 4). Among the analyzed collective of patients, patients suffering from PNS disorders had comparatively low mortality rates (OR: 0.25, 95% CI 0.09–0.66).

**Table 4:** Predictors of mortality

Parameter	A. Influence of disease categories on mortality: multivariable full model					B. Influence of diseases on mortality: multivariable full model			
	Odds ratio	2.5% CI	97.5% CI	p		Odds ratio	2.5% CI	97.5% CI	p
(Intercept)	0.00	0.00	0.00	< 0.001	(Intercept)	0.00	0.00	0.00	< 0.001
Cerebrovascular disorder	8.13	3.83	17.26	< **0.001**	Acute ischemic stroke	3.89	1.85	8.17	< **0.001**
Neuromuscular disorder	0.25	0.09	0.66	**0.005**	Intracerebral hemorrhage	6.14	2.53	14.89	< **0.001**
Encephalopathy	1.29	0.65	2.53	0.466	CIP/CIM	0.21	0.08	0.58	**0.003**
Inflammatory CNS disorder	0.79	0.04	14.73	0.875	Delirium	0.79	0.37	1.67	0.540
Epileptic disorder	0.77	0.28	2.08	0.607	Inflammatory CNS disorder	0.81	0.05	12.20	0.882
					Epileptic disorder	1.01	0.38	2.69	0.981
Age (years)	1.06	1.03	1.10	< **0.001**		1.07	1.04	1.11	< **0.001**
Female sex	0.88	0.45	1.70	0.702		0.73	0.37	1.44	0.358
Preexisting neurological diseases	0.62	0.28	1.36	0.229		0.59	0.27	1.27	0.174
Preexisting cardiovascular diseases	1.24	0.57	2.72	0.584		1.27	0.59	2.77	0.538
Vasopressors	6.91	1.81	26.41	**0.005**		6.91	1.84	25.91	**0.004**
Invasive ventilation	1.07	0.30	3.78	0.921		1.03	0.29	3.67	0.958
ARDS^a^	1.30	0.54	3.16	0.556		1.29	0.54	3.10	0.561
Acute kidney failure	1.39	0.67	2.88	0.373		1.43	0.69	2.97	0.331
Acute liver failure	4.21	1.60	11.05	**0.004**		4.25	1.63	11.11	**0.003**
Sepsis	1.69	0.82	3.48	0.153		1.41	0.69	2.87	0.348
ECMO^a^	2.48	1.05	5.85	**0.037**		2.68	1.15	6.23	**0.022**
pmRS (points)^b^	1.37	1.07	1.75	**0.012**		1.44	1.12	1.85	**0.005**
LEOSS stage of disease: complicated^c^	1.33	0.47	3.82	0.589		1.14	0.43	3.06	0.792
LEOSS stage of disease: critical^c^	1.59	0.57	4.48	0.375		1.46	0.52	4.10	0.471
Anticoagulant drugs on admission	2.35	1.11	4.96	**0.026**		2.00	0.95	4.21	0.068
Time from COVID-19 diagnosis to first neurologic deficit (days)^b^	0.97	0.95	1.00	**0.027**		0.97	0.95	1.00	**0.025**

Bold values are significant (*p* < 0.05)

A: The influence of categories of disease on mortality was examined in a multivariable logistic regression model adjusted for the variables (age, sex, preexisting neurological disease, preexisting cardiovascular disease, vasopressors, invasive ventilation, ARDS, acute kidney failure, acute liver failure, sepsis, ECMO, pmRS, LEOSS stage of disease complicated and critical, anticoagulant drugs on admission, duration of COVID-19 diagnosis until first neurologic deficit

B: The influence of the most common diseases (like acute ischemic stroke, intracerebral hemorrhage, CIP/CIM and Delirium) on mortality was examined in a multivariable logistic regression model adjusted for the same variables as in Table 4A

ICH: intracerebral hemorrhage, CIP/CIM: critical illness polyneuropathy/myopathy, ARDS: acute respiratory distress syndrome, CNS: central nervous system, ECMO: extracorporeal membrane oxygenation

^a^88/91 ECMO patients had ARDS. The ECMO variable might act as an indicator for severe ARDS, whereas ARDS without ECMO is likely not as severe

^b^Age/Duration/mRS were used as continuous covariates; odds ratios are given per point on the respective scale. To get, for example, the estimated odds ratio of 10 years of age, calculate 1.02^(10)

^c^Compared to LEOSS Stage uncomplicated

## Discussion

Our study provides new insights into prevalence, clinical characteristics and predictors of mortality in COVID-19 ICU patients with neurological manifestations. Overall, 12.7% of all COVID-19 ICU patients admitted to ICUs of the study centers during a 17-month period developed neurologic manifestations. The most common ones were encephalopathic, cerebrovascular and neuromuscular disorders, with delirium, AIS, ICH and CIP/CIM being the most prevalent diagnoses. There was hardly any evidence of COVID-related encephalitis. Overall, the occurrence of any cerebrovascular disorder was the strongest predictor for death. Cerebrovascular complications were associated with an unfavorable outcome, i.e., ICH with a 6.1-fold and AIS with a 3.9-fold increase in in-hospital mortality.

In comparison with other studies in this field, our study is the only one to focus on neurologic manifestations of COVID-19 in the ICU exclusively, while other studies evaluated broader cohorts, used different definitions of disorders and frequently did not involve neurologists for assessment. Most studies not limited to ICU patients reported higher frequencies of neurological manifestations [7, 9, 11, 15], which might be explained by inclusion of milder and/or rather non-specific symptoms like headache, anosmia or fatigue. As many ICU patients present with an altered mental status, some neurologic symptoms may have stayed undetected. The baseline and clinical characteristics of the PANDEMIC cohort are quite similar to previously described COVID-19 ICU cohorts not focusing on neurologic manifestations [1, 3, 7, 20, 21]. Hence, we assume that our results are transferable to other ICU populations. The prevalence as reported here is in line with a report by Frontera et al. describing neurological manifestations in 13.6% of all hospitalized patients [22]. Although their study involved 78% (3,504/4,492) non-ICU patients, they captured a similar spectrum of manifestations compared to our study. In another prospective registry study focusing on non-ICU COVID patients (4.1% ICU patients), using similar categories of neurological manifestations (cerebrovascular diseases, encephalopathy, seizures and meningoencephalitis), the authors calculated a prevalence of 12.9% [12]. A retrospective study by Helms et al*.* [6] on COVID-19-ICU patients found neurologic manifestations, mostly agitation, delirium, or other types of encephalopathy, in 67% (39/58) of patients after withdrawal of sedation. The differences might be related to different timepoints of assessment as well as to the fact that only ARDS patients were assessed and no non-neurologic “control” population; hence, the rate can hardly be regarded a prevalence. Older patients with more severe COVID-19 disease seemed to have an increased risk of neurologic manifestations. This is in line with findings of higher SOFA scores by Frontera et al. in patients with neurologic manifestations and the study of Kleineberg et al. who found more severe neurologic complications in patients reaching the critical or complicated LEOSS stage of disease (complicated stage: supplemental oxygen necessary; critical stage: use of mechanical ventilation, dialysis and/or catecholamines) [7, 9].

The total number of cerebrovascular events was comparable to previously reported ICU COVID-19 cases, but we found a higher prevalence of AIS in our ICU cohort (3.5%) compared to previous studies in broader cohorts (0.04–1.9%) [7, 9, 12, 23, 24]. A large population-based study in Sweden found a 2.1- to 6.2-fold increased risk for AIS among COVID-19 patients [24]. In the large European LEOSS-registry, a correlation between increased prevalence of AIS and COVID-19 stage of disease was demonstrated, with COVID-19-associated coagulopathy as a hypothesis [7]. However, previous studies in critically ill patients with diseases other than COVID-19 such as sepsis and ARDS, found similar rates of AIS [25–32]. Hence, the proposed mechanisms such as pro-coagulatory inflammation or endotheliitis on the complication AIS remains questionable, as well as the order of events.

Concerning hemorrhagic stroke, the prevalence we found (3.0%; with ICH 1.8%, and SAH 1.2%) was higher compared to most previous studies in broader hospitalized COVID-19 cohorts [33–36]. Yet, Kleineberg et al. [7] reported a prevalence of even 5% in patients at the critical stage. In their and our cohort ECMO and higher aPTT values were associated with a higher risk of ICH, which appears plausible [7]. A study based on a propensity matched non-COVID cohort could not find an increased rate in hemorrhagic stroke among COVID patients [37]. Moreover, ICH rates in other ICU cohorts including ECMO are in the same range. Hence, again, it remains unclear whether COVID-19 is an independent risk factor for hemorrhagic stroke or whether this is a consequence of the aggressive ICU treatment [18, 25, 26, 32].

The frequency of non-vascular neurologic manifestations as encephalopathic, epileptic or neuromuscular disorders shows a large variability in the preexisting literature, depending on timepoint of assessment, qualification of the investigator, definitions of disorders and control for confounders. According to our data and in line with previous reports, no specific direct pathomechanism between those manifestations and COVID-19 became obvious [19, 38]. The number of patients with positive PCR detection of the virus in CSF or brain tissue was negligible which supports previous results arguing against SARS-CoV-2-associated meningoencephalitis [7, 39, 40].

Overall ICU mortality in the preexisting literature on COVID-19 varies greatly depending on the geographic location, the period of analysis and the clinical characteristics of the analyzed cohort and ranges between 21 and 100% [41–43]. A German study performed in 2020 in ventilated patients reported a mortality of 38.8% [44]. Another earlier study confirmed differences in mortality depending on age, sex and comorbidities [45]. Based on a review article by Misra et al., a higher mortality in COVID-19-ICU patients with neurologic complications can be expected [15]. This was also in line with registry data of 16,225 COVID-19 patients suggesting higher odds for death and worse functional outcome in patients with neurologic manifestations [12]. Total in-hospital mortality in our preselected ICU cohort appears within the range reported in the literature. Possibly, neurologic manifestations in previously published ICU cohorts contributed to mortality, but remained undetected being masked by the clinical course being dominated by respiratory failure. Our data suggest that neurologic manifestations as a predictor of mortality differ considerably. For instance, some patients get diagnosed with CIP/CIM later in their ICU course, often after having survived the critical COVID-19 phase. On the other hand, patients with cerebrovascular complications show a much higher mortality of almost 60%, which is in line with the current literature [9, 11]. Thus, the diverse neurological manifestations in COVID-19 ICU patients may give rise to different modes of prognostication, decision-making, triage and (preventive or symptomatic) treatment.

Unfavorable outcomes at ICU discharge were noticed in a higher number of patients (mortality 36%, only 17.7% could be discharged in home care, mRS 3–5; in 67.9% of the survivors) compared to other studies, reflecting the prediction of worse outcome by some neurological diagnosis. In addition to long-term effects of COVID-19 (post-COVID-19 syndrome), ICU survivors were described to suffer an even lower quality of life due to persistent fatigue, dyspnea, sleep disturbances, and mental health issues [46]. Similarly, post-intensive care syndrome (PICS) has been described after shock, sepsis, hypoxia, ARDS or delirium, significantly increasing the risk of long-term cognitive and physical impairment [47, 48]. Longer-term studies with follow-up and control groups are warranted to shed light on this aspect [49–51].

Our study has several limitations. Some of these are related to a pragmatic study design allowing for data collection by ICU physicians during a pandemic with uncertain course. First, and inherent to the ICU cohort, neurologic findings could have been missed due to sedation or other ICU measures, failure to consult a neurologist with ICU experience or difficulties performing adequate diagnostic investigations. Thus, the prevalence of neurologic manifestations might have been underestimated. Another potential compromise regarding prevalence is due to the aspect that the “control” group of those COVID-19 ICU patients without detected neurologic abnormalities was not further characterized and was not included in our regression model, which has certainly caused ascertainment bias. Moreover, the multicenter approach might increase the degree of heterogeneity. However, all sites used a standardized eCRF with precise directives and definitions to minimize variability. In addition, the electronic record was combined with a plausibility check and followed by data clearing. Another possible limitation could be associated with the inclusion period of the study (between April 2020 and September 2021), in which more than one variants of SARS-COV-2 caused at least two waves. Possible differences concerning neurologic manifestations or outcomes of different variants might have been missed. Nevertheless, a subgroup analysis comparing patients included during the first wave (April 2020–August 2021) versus patients included during the second wave (August 2021–September 2021) did not reveal any significant differences regarding prevalence or type of occurred neurological manifestations (data not shown).

Strengths of our study are its prospective nature, the focus on ICU patients as a distinct yet very relevant COVID-19 subgroup, use of a standardized eCRF, employment of consultations by experienced neurointensivists, and a large sample size given that special subgroup yielded by a multicenter approach reflecting a real-world scenario.

## Conclusion

In the prospective PANDEMIC registry study, we provide a detailed description of the neurologic symptoms and diagnoses of critically ill COVID-19 patients. Neurological manifestations were reported in 12.7% of COVID-19 ICU patients. The most common ones were encephalopathy, cerebrovascular and neuromuscular disorders, with delirium acute ischemic stroke, intracranial hemorrhage and critical illness polyneuropathy/myopathy being the most prevalent underlying diagnoses. Of these, AIS and ICH were most strongly associated with higher morbidity and mortality. COVID-related encephalitis was not noteworthy in our study. Hence, a direct effect by SARS-CoV-2 on the majority of neurologic manifestations remains questionable. Uniform protocols, prospective screening and long-term follow-up are warranted to better understand the impact of ICU treatment in neurologically compromised COVID-19 patients.

## Supplementary Information

**Additional file 1.** Additional information on Methods (list of parameters listed in the eCRF, list of categories of disorders and underlying diseases, definitions and statistics. **Supplement Table 1**: Neurologic past medical history according to disease categories (multiple answers possible). **Supplement Table 2**: PANDEMIC inclusion criteria and COVID state of disease according to disease categories (multiple answers possible). **Supplement Table 3**: Neurologic symptoms according to disease categories in 392 patients (multiple answers possible). **Supplement Table 4**: Diagnostic findings in 392 patients (multiple answers possible). **Supplement Table 5**: Factors influencing the occurrence of a disease category. **Supplement Table 6**: Factors influencing the occurrence of most common diseases. **Supplement Table 7**: Factors influencing the decision to therapy limitation.

## Data Availability

All data generated or analyzed during this study are included in this published article [and its supplementary information files].

## Abbreviations

AIS: Acute ischemic stroke
ARDS: Acute respiratory distress syndrome
CI: Confidence interval
CIP/CIM: Critical illness polyneuropathy/myopathy
CNS: Central nervous system
COVID: Coronavirus disease
CSF: Cerebrospinal fluid
CV: Cerebrovascular disorder
DGNI: German Society for Neurologic Intensive Care and Emergency Medicine
ECMO: Extracorporeal membrane oxygenation
eCRF: Electronic case report form
FOUR: Full Outline of UnResponsiveness
GCS: Glasgow Coma Scale
ICH: Intracerebral hemorrhage
ICU: Intensive care unit
IGNITE: Initiative of German NeuroIntensive Trial Engagement
NMD: Neuromuscular disorder
mRS: Modified Ranking Scale
OR: Odds ratio
PANDEMIC: Pooled Analysis of Neurologic DisordErs Manifesting in Intensive care of COVID-19
PCR: Polymerase chain reaction
RASS: Richmond Agitation-Sedation Scale
SAH: Subarachnoid hemorrhage
SAPS: Simplified Acute Physiology Score
SARS-CoV: Severe acute respiratory syndrome coronavirus 2
SOFA: Sequential Organ Failure Assessment Score
