# Supplementary material for: Neurologic manifestations of COVID-19 in critically ill patients: results of the prospective multicenter registry PANDEMIC
Source: Crit Care. 2022 Jul 16;26:217. doi: 10.1186/s13054-022-04080-3 (PMC9287707; doi:10.1186/s13054-022-04080-3)
Supplement: Supplementary file 1 — Additional file 1. Additional information on Methods (list of parameters listed in the eCRF, list of categories of disorders and underlying diseases, definitions and statistics. Supplement Table 1: Neurologic past medical history according to disease categories (multiple answers possible). Supplement Table 2: PANDEMIC inclusion criteria and COVID state of disease according to disease categories (multiple answers possible). Supplement Table 3: Neurologic symptoms according to disease categories in 392 patients (multiple answers possible). Supplement Table 4: Diagnostic findings in 392 patients (multiple answers possible). Supplement Table 5: Factors influencing the occurrence of a disease category. Supplement Table 6: Factors influencing the occurrence of most common diseases. Supplement Table 7: Factors influencing the decision to therapy limitation. [file 13054_2022_4080_MOESM1_ESM.docx]

Title:

Neurologic manifestations of COVID-19 in critically ill patients: results of the prospective multicenter registry PANDEMIC.

Journal: Intensive Care Medicine

Corresponding Author:

Konstantinos Dimitriadis MD, ORCID: 0000-0003-0417-2267, Institute for Stroke and Dementia Research (ISD), Ludwig-Maximilians-Universität (LMU), Munich, Germany, Feodor-Lynen-Straße 17, 81377 Munich, Germany. Tel ++49-89-4400-46048, [Konstantin.Dimitriadis@med.uni-muenchen.de](mailto:Konstantin.Dimitriadis@med.uni-muenchen.de),

PANDEMIC: **P**ooled **A**nalysis of **N**eurologic **D**isord**E**rs **M**anifesting in **I**ntensive care of **C**OVID-19

|  |
| --- |

**Methods:**

**Parameters recorded in eCRF:**

Recorded parameters included demographic data, past medical history, premorbid mRS ((pmRS) score; a 7-point scale reflecting daily functioning ranging from 0 (full independence without symptoms) to 6 (death)), ICU course of disease, laboratory tests including cerebrospinal fluid analysis, results of neurophysiological and neuroradiological diagnostics (electroencephalography (EEG), magnet resonance imaging (MRI), nerve conduction tests (ENG or NLG), evoked potentials (EP)). Outcome measures included mRS at discharge, in-hospital death, discharge destination, scores grading the severity of critical illness such as Sequential Organ Failure Assessment (SOFA) score and Simplified Acute Physiology Score (SAPS), the sedation score Richmond Agitation-Sedation Scale (RASS), scores reflecting level of consciousness such as Glasgow Coma Scale (GCS) and Full Outline of UnResponsivness (FOUR) score, and the level of suspicion for “Neuro-COVID” by the consulting neurologist. Data were collected by the neurologist by examination or chart review, pseudonymized and entered into a secure electronic case report form (eCRF) (RedCap database). In addition, disease stages of COVID-19 were documented on admission and during the clinical course. These were defined according to the LEOSS registry (Lean European Open Survey for SARS-CoV-2 Infected Patients): 1. uncomplicated phase without symptoms or slight symptoms of upper respiratory tract, fever or diarrhea; 2. complicated phase when patients required oxygen supplementation; 3. critical phase involving mechanical ventilation, dialysis and/or catecholamines^7,21^.

**Categories of disorders and underlying diagnoses**

Since the nature and diversity of neurologic complications for COVID-19 ICU patients were unclear at the start of the study and in order not to miss relevant manifestations, we used an inductive process to define categories of disease by using all available data.

Categories of disease with subcategories:

1. Cerebrovascular disorder (CV): acute ischemic stroke (AIS), intracerebral hemorrhage (ICH), subarachnoid hemorrhage (SAH), cerebral venous and sinus thrombosis (CVST), subdural hemorrhage (SDH), posterior reversible encephalopathy syndrome (PRES)/ reversible cerebral vasoconstriction syndrome (RCVS) and microbleeds.
2. Neuromuscular disorder (NMD-Disorder): critical illness polyneuropathy or myopathy (CIP/CIM), Guillain-Barré Syndrome (GBS), Miller Fisher Syndrome (MFS), myositis and myasthenia.
3. Encephalopathy: non-hypoxic encephalopathy, hypoxic encephalopathy, delirium, disorder of consciousness with unknown etiology,
4. Inflammatory central nervous system disorder (CNS): encephalitis, meningitis, myelitis, meningoencephalitis, herpes zoster oticus.
5. Epileptic disorder: seizures and status epilepticus.
6. Other: olfactory impairment, brain edema, benign paroxysmal positional vertigo (BPPV), peripheral facial palsy, plexus lesion, fine motor impairment not further specified, gaze palsy not further specified, tetraparesis not further specified, phobic gait disorder, major depression and restless legs syndrome (RLS).

Definitions:

Delirium was defined according to the Diagnostic and Statistical Manual of Mental Disorders, 5th Edition (DSM 5), as a clinical syndrome comprising acute disturbances in attention and awareness. Diagnosis was on the ICDCS or the CAM-ICU Scale.

Encephalopathy was defined according to the National Institute of Neurological Disorders and Stroke (NINDS) as a diffuse disease of the brain that alters brain function or structure. This category was used based on clinical criteria or electroencephalographic criteria.

Impaired consciousness was used for a spectrum of states: coma (based on clinical criteria or CGCS<9), akinetic mutism, minimally conscious state, vegetative state and brain death, acknowledging that the listed conditions go along with impaired consciousness, but the latter (alone) is not sufficient to define the conditions.

**Statistics:**

Explanatory variables included in the multivariable models (depending on the respective research question): age, sex, pre-existing neurological diseases, pre-existing cardiovascular diseases, vasopressors, invasive ventilation, ARDS, acute kidney failure, acute liver failure, sepsis, extracorporeal membrane oxygenation (ECMO), pmRS, LEOSS stage of disease, anticoagulant drugs on admission, duration of COVID diagnosis until first neurologic deficit, hypertension, nicotine consumption, dyslipidemia, diabetes mellitus, ischemic stroke in past medical history, anticoagulant drugs on admission, ischemic stroke in past medical history, acute ischemic stroke, platelet count, aPTT, INR, IL6, SAPS score, time from ICU admission to first neurologic symptoms, muscle relaxant drugs, dementia in past medical history and alcohol consumption. Missing data: the number of included data sets per variable are provided in all tables presenting descriptive data.

**Supplement Table 1: Neurologic past medical history according to disease categories (multiple answers possible)**

|  | **CV disorder** | **Neuromuscular disorder** | **Encephalo- pathy** | **Infl. CNS disorder** | **Epileptic disorder** | **Other** | **Total** |
| --- | --- | --- | --- | --- | --- | --- | --- |
|  |  |  |  |  |  |  |  |
| Any of the ones below (N= 386) | 47 (29.7%) | 14 (17.5%) | 43 (24.3%) | 2 (28.6%) | 11 (28.2%) | 11 (18.3%) | 88 (22.8%) |
| Acute ischemic stroke | 22 (13.9%) | 1 (1.3%) | 14 (7.9%) | 0 (0.0%) | 4 (10.3%) | 2 (3.3%) | 33 (8.5%) |
| Hemorrhagic stroke | 0 (0.0%) | 1 (1.3%) | 1 (0.6%) | 0 (0.0%) | 1 (2.6%) | 0 (0.0%) | 2 (0.5%) |
| Subarachnoid hemorrhage | 0 (0.0%) | 0 (0.0%) | 2 (1.1%) | 0 (0.0%) | 0 (0.0%) | 0 (0.0%) | 2 (0.5%) |
| Traumatic brain injury | 0 (0.0%) | 0 (0.0%) | 2 (1.1%) | 0 (0.0%) | 1 (2.6%) | 0 (0.0%) | 2 (0.5%) |
| Dementia | 5 (3.2%) | 2 (2.5%) | 7 (4.0%) | 0 (0.0%) | 1 (2.6%) | 0 (0.0%) | 12 (3.1%) |
| Epileptic disorder | 5 (3.2%) | 1 (1.3%) | 8 (4.5%) | 0 (0.0%) | 5 (12.8%) | 3 (5.0%) | 12 (3.1%) |
| Idiopathic Parkinson´s disease | 1 (0.6%) | 1 (1.3%) | 3 (1.7%) | 0 (0.0%) | 0 (0.0%) | 0 (0.0%) | 3 (0.8%) |
| Multiple sclerosis | 0 (0.0%) | 0 (0.0%) | 0 (0.0%) | 0 (0.0%) | 0 (0.0%) | 0 (0.0%) | 0 (0.0%) |
| Myasthenia gravis | 0 (0.0%) | 1 (1.3%) | 1 (0.6%) | 0 (0.0%) | 0 (0.0%) | 0 (0.0%) | 2 (0.5%) |
| Other^a^ | 23 (14.6%) | 8 (10.0%) | 16 (9.0%) | 2 (28.6%) | 3 (7.7%) | 6 (10.0%) | 39 (10.1%) |
|  |  |  |  |  |  |  |  |

a Other included: polyneuropathy, sinus thrombosis, carotid artery dissection, subdural hematoma, encephalitis, cerebelitis, depression, traumatic spinal injury, brain tumor, GBS, syringomyelia, restless legs syndrom and others.

**Supplement Table 2: PANDEMIC inclusion criteria and COVID state of disease according to disease categories (multiple answers possible)**

|  | **CV disorders** | **Neuromuscular disorder** | **Encephalo- pathy** | **Infl. CNS disorders** | **Epileptic disorders** | **Other** | **Total** |
| --- | --- | --- | --- | --- | --- | --- | --- |
| **Breakdown of inclusion criteria in 392 patients (at least one had to be fulfilled)** |  |  |  |  |  |  |  |
| New neurological manifestations (N=375) | 148 (93.7%) | 66 (95.7%) | 153 (86.4%) | 7 (100.0%) | 35 (89.7%) | 58 (95.1%) | 339 (90.4%) |
| New psychiatric symptoms (N=347) | 7 (4.8%) | 16 (24.2%) | 50 (30.5%) | 0 (0.0%) | 6 (15.8%) | 3 (5.4%) | 61 (17.6%) |
| COVID-19 test in CSF/brain tissue performed and positive ^a^ | 1 | 1 | 1 | 1 | 0 | 0 | 3 |
| Neurological consultation (N=396) | 120 (77.9%) | 77 (97.5%) | 116 (67.1%) | 7 (100.0%) | 36 (90.0%) | 37 (61.7%) | 275 (72.8%) |
|  |  |  |  |  |  |  |  |
| LEOSS stage of disease on admission (N=291) |  |  |  |  |  |  |  |
| - uncomplicated | 28 (23.5%) | 6 (10.5%) | 26 (18.2%) | 1 (16.7%) | 4 (13.3%) | 2 (4.1%) | 49 (16.8%) |
| - complicated | 30 (25.2%) | 12 (21.1%) | 53 (37.1%) | 0 (0.0%) | 13 (43.3%) | 17 (34.7%) | 101 (34.7%) |
| - critical | 61 (51.3%) | 39 (68.4%) | 64 (44.8%) | 5 (83.3%) | 13 (43.3%) | 30 (61.2%) | 141 (48.5%) |

| a A negative answer to this question can either mean that a test was not performed or that it was negative. Most of the centers did not perform CSF PCR tests. The true number of tests that was performed is unknown. |
| --- |

**Supplement Table 3: Neurologic symptoms according to disease categories in 392 patients (multiple answers possible)**

|  | **CV disorders** | **Neuromuscular disorder** | **Encephalo- pathy** | **Infl. CNS disorders** | **Epileptic disorders** | **Other** | **Total** |
| --- | --- | --- | --- | --- | --- | --- | --- |
|  |  |  |  |  |  |  |  |
| Ageusia | 1 (0.6%) | 1 (1.3%) | 2 (1.1%) | 0 (0.0%) | 0 (0.0%) | 14 (23.0%) | 14 (3.6%) |
| Anosmia | 2 (1.2%) | 1 (1.3%) | 3 (1.7%) | 0 (0.0%) | 0 (0.0%) | 14 (23.0%) | 14 (3.6%) |
| Headache | 0 (0.0%) | 1 (1.3%) | 2 (1.1%) | 0 (0.0%) | 0 (0.0%) | 2 (3.3%) | 5 (1.3%) |
| Vertigo | 3 (1.9%) | 1 (1.3%) | 4 (2.2%) | 0 (0.0%) | 0 (0.0%) | 1 (1.6%) | 6 (1.5%) |
| Impaired consciousness | 80 (49.7%) | 41 (51.3%) | 154 (85.1%) | 4 (57.1%) | 24 (60.0%) | 23 (37.7%) | 228 (58.2%) |
| Motor deficit | 93 (57.8%) | 70 (87.5%) | 54 (29.8%) | 7 (100.0%) | 13 (32.5%) | 21 (34.4%) | 187 (47.7%) |
| Speech disorder | 54 (33.5%) | 5 (6.3%) | 19 (10.5%) | 1 (14.3%) | 5 (12.5%) | 3 (4.9%) | 66 (16.8%) |
| Sensory deficit | 28 (17.4%) | 18 (22.5%) | 17 (9.4%) | 2 (28.6%) | 5 (12.5%) | 3 (4.9%) | 50 (12.8%) |
| Epileptic seizure | 14 (8.7%) | 2 (2.5%) | 17 (9.4%) | 0 (0.0%) | 36 (90.0%) | 1 (1.6%) | 36 (9.2%) |
| Other Symptoms^a^ | 70 (43.5%) | 22 (27.5%) | 49 (27.1%) | 4 (57.1%) | 12 (30.0%) | 24 (39.3%) | 119 (30.4%) |
|  |  |  |  |  |  |  |  |

a Other Symptoms include: Anisocoria, Areflexia, Tendency to fall, Ataxia, Agitation, Hallucinations.

**Supplement Table 4: Diagnostic findings in 392 patients (multiple answers possible)**

|  | **Number of Examinations (Nr. of Patients)^a^** | **Number of pathological Examinations (Nr. of Patients)^a^** | **Main Findings Number of Examinations (Nr. of Patients)^a^** |
| --- | --- | --- | --- |
| Cranial computer tomography | 409 (264) | 258 (158) | Acute ischemic stroke: 116 (80) |
|  |  |  | Intracerebral hemorrhage: 84 (47) |
|  |  |  | Subarachnoid hemorrhage: 55 (27) |
|  |  |  | Global cerebral edema: 29 (22) |
|  |  |  |  |
| Magnetic resonance imaging | 81 (71) | 64 (55) | Acute ischemic stroke: 24 (21) |
|  |  |  | Intracerebral hemorrhage: 6 (5) |
|  |  |  | Subarachnoid hemorrhage: 5 (5) |
|  |  |  | Microbleeds: 9 (9) |
|  |  |  | Leucencephalopathy: 18 (18) |
|  |  |  |  |
| Electroencephalography | 75 (62) | 66 (54) | Encephalopathy: 47 (38) |
|  |  |  | Periodic pattern: 4 (3) |
|  |  |  | Epileptiform discharges: 8 (6) |
|  |  |  | Status epilepticus: 3 (2) |
|  |  |  |  |
| Electroneuro-/myography | 25 (21) | 23 (20) | Demyelination: 8 (7) |
|  |  |  | Axonal degeneration: 19 (17) |
|  |  |  | Signs of denervation: 8 (7) |
|  |  |  |  |
| Evoked potentials | 7 (7) | 5 (5) | Pathologic findings: 5 (5) |
|  |  |  |  |
| Transcranial doppler sonography | 33 (31) | 13 (13) | Macroangiopahty 13 (13) |

a some patients were examined more than once.

| Table 14: Aufenthaltsdauer |
| --- |

**Supplement Table 5: Factors influencing the occurrence of a disease category**

**a**

|  | **Cerebrovascular disorder** | | | | **Neuromuscular disorder** | | | |
| --- | --- | --- | --- | --- | --- | --- | --- | --- |
| **Parameter** | **Odds-ratio** | **2.5% CI** | **97.5% CI** | **p** | **Odds-ratio** | **2.5% CI** | **97.5% CI** | **p** |
| (Intercept) | 0.35 | 0.07 | 1.70 | 0.192 | 0.12 | 0.02 | 0.97 | 0.047 |
| Age (years) ^a^ | 1.02 | 1.00 | 1.04 | 0.045 | 0.99 | 0.96 | 1.02 | 0.425 |
| Female sex | 0.84 | 0.50 | 1.40 | 0.502 | 1.01 | 0.53 | 1.91 | 0.983 |
| Pre-existing neurological diseases | 1.61 | 0.88 | 2.95 | 0.121 | 0.90 | 0.40 | 2.02 | 0.792 |
| Pre-existing cardiovascular diseases | 0.68 | 0.39 | 1.21 | 0.191 | 0.89 | 0.47 | 1.70 | 0.720 |
| Vasopressors | 0.96 | 0.33 | 2.79 | 0.947 | 0.29 | 0.07 | 1.27 | 0.100 |
| Invasive ventilation | 3.08 | 1.06 | 8.95 | 0.039 | 3.17 | 0.63 | 15.9 | 0.160 |
| ARDS^b^ | 0.25 | 0.13 | 0.50 | <0.001 | 2.06 | 0.79 | 5.33 | 0.138 |
| Acute kidney failure | 0.85 | 0.48 | 1.51 | 0.578 | 0.65 | 0.33 | 1.28 | 0.208 |
| Acute liver failure | 2.86 | 1.36 | 6.03 | 0.006 | 0.53 | 0.20 | 1.40 | 0.201 |
| Sepsis | 0.72 | 0.41 | 1.26 | 0.248 | 1.09 | 0.55 | 2.14 | 0.801 |
| ECMO^b^ | 3.75 | 1.97 | 7.11 | <0.001 | 1.23 | 0.62 | 2.46 | 0.548 |
| pmRS (points) ^a^ | 1.00 | 0.83 | 1.20 | 0.995 | 1.09 | 0.87 | 1.37 | 0.460 |
| LEOSS stage of disease: complicated^c^ | 0.45 | 0.20 | 1.04 | 0.061 | 0.82 | 0.26 | 2.65 | 0.741 |
| LEOSS stage of disease: critical^c^ | 0.84 | 0.36 | 1.97 | 0.693 | 1.83 | 0.58 | 5.83 | 0.302 |
| Anticoagulant Drugs on admission | 1.13 | 0.59 | 2.14 | 0.712 | 0.85 | 0.37 | 1.95 | 0.698 |
| Time of COVID diagnosis to first neurologic deficit (days)^a^ | 0.98 | 0.96 | 1.00 | 0.018 | 1.04 | 1.02 | 1.06 | <0.001 |

**b**

|  | **Encephalopathy** | | | | **Epileptic disorders** | | | |
| --- | --- | --- | --- | --- | --- | --- | --- | --- |
| **Parameter** | **Odds-ratio** | **2.5% CI** | **97.5% CI** | **p** | **Odds-ratio** | **2.5% CI** | **97.5% CI** | **p** |
| (Intercept) | 0.58 | 0.13 | 2.55 | 0.468 | 1.22 | 0.14 | 10.8 | 0.855 |
| Age (years) ^a^ | 1.01 | 0.98 | 1.03 | 0.627 | 0.96 | 0.93 | 0.99 | 0.014 |
| Female sex | 0.94 | 0.58 | 1.51 | 0.789 | 1.24 | 0.58 | 2.63 | 0.576 |
| Pre-existing neurological diseases | 1.09 | 0.61 | 1.95 | 0.779 | 1.22 | 0.51 | 2.95 | 0.654 |
| Pre-existing cardiovascular diseases | 1.06 | 0.63 | 1.78 | 0.835 | 0.82 | 0.35 | 1.90 | 0.638 |
| Vasopressors | 2.78 | 0.91 | 8.51 | 0.074 | 1.58 | 0.31 | 8.07 | 0.585 |
| Invasive ventilation | 0.26 | 0.09 | 0.81 | 0.020 | 0.42 | 0.08 | 2.10 | 0.292 |
| ARDS^b^ | 2.32 | 1.21 | 4.44 | 0.012 | 0.55 | 0.20 | 1.51 | 0.243 |
| Acute kidney failure | 1.59 | 0.94 | 2.69 | 0.085 | 1.68 | 0.72 | 3.96 | 0.231 |
| Acute liver failure | 0.79 | 0.39 | 1.59 | 0.506 | 0.75 | 0.22 | 2.56 | 0.643 |
| Sepsis | 1.29 | 0.76 | 2.18 | 0.342 | 1.79 | 0.73 | 4.36 | 0.201 |
| ECMO^b^ | 0.42 | 0.23 | 0.75 | 0.004 | 0.82 | 0.32 | 2.13 | 0.689 |
| pmRS (points) ^a^ | 1.05 | 0.87 | 1.27 | 0.609 | 1.15 | 0.87 | 1.51 | 0.322 |
| LEOSS stage of disease: complicated^c^ | 0.83 | 0.40 | 1.74 | 0.622 | 1.76 | 0.51 | 6.02 | 0.369 |
| LEOSS stage of disease: critical^c^ | 0.53 | 0.24 | 1.18 | 0.119 | 1.24 | 0.30 | 5.23 | 0.765 |
| Anticoagulant Drugs on admission | 0.86 | 0.47 | 1.56 | 0.618 | 1.41 | 0.53 | 3.74 | 0.490 |
| Time of COVID diagnosis to first neurologic deficit (days)^a^ | 1.00 | 0.98 | 1.01 | 0.661 | 0.99 | 0.96 | 1.01 | 0.344 |

a Age/Duration/mRS were used as continuous covariates, odds ratios are given per point on the respective scale. To get, for example, the estimated odds ratio of 10 years of age, calculate 1.02^(10).

b 88/91 ECMO patients had ARDS. The ECMO variable might act as an indicator for severe ARDS, whereas ARDS without ECMO is likely not as severe.

c Compared to LEOSS Stage uncomplicated

ECMO: Extracorporeal membrane oxygenation, ARDS: Acute respiratory distress Syndrome, pmRS: premorbide modified Ranking Scale.

**Supplement Table 6: Factors influencing the occurrence of most common diseases**

**A.**

|  | **Acute ischemic stroke** | | | |
| --- | --- | --- | --- | --- |
| **Parameter** | **Oddsratio** | **2.5% CI** | **97.5% CI** | **p** |
| (Intercept) | 0.12 | 0.02 | 0.63 | 0.012 |
| Age (years)^a^ | 1.03 | 1.01 | 1.05 | 0.010 |
| Female sex | 0.91 | 0.52 | 1.59 | 0.741 |
| Hypertension | 0.59 | 0.32 | 1.06 | 0.076 |
| Nicotine consumption | 0.52 | 0.22 | 1.25 | 0.142 |
| Dyslipidemia | 1.73 | 0.86 | 3.47 | 0.123 |
| Diabetes mellitus | 1.40 | 0.75 | 2.60 | 0.288 |
| Ischemic stroke in past medical history | 3.66 | 1.51 | 8.88 | 0.004 |
| Anticoagulant drugs on admission | 0.94 | 0.48 | 1.84 | 0.866 |
| LEOSS stage of disease: complicated^b^ | 0.42 | 0.20 | 0.89 | 0.024 |
| LEOSS stage of disease: critical^b^ | 0.45 | 0.22 | 0.95 | 0.037 |
| ECMO | 0.86 | 0.43 | 1.71 | 0.670 |

**B.**

|  | **intracerebral hemorrhage (ICH)** | | | | **Haemorrhagic stroke (ICH+SAH)** | | | |
| --- | --- | --- | --- | --- | --- | --- | --- | --- |
| **Parameter** | **Odds-ratio** | **2.5% CI** | **97.5% CI** | **p** | **Odds-ratio** | **2.5% CI** | **97.5% CI** | **p** |
| (Intercept) | 0.05 | 0.00 | 0.72 | 0.027 | 0.08 | 0.01 | 0.82 | 0.033 |
| Age (years) ^a^ | 0.99 | 0.96 | 1.02 | 0.519 | 1.00 | 0.97 | 1.03 | 0.880 |
| Female sex | 1.33 | 0.67 | 2.67 | 0.415 | 0.92 | 0.48 | 1.78 | 0.807 |
| Hypertension | 1.05 | 0.50 | 2.21 | 0.898 | 0.88 | 0.45 | 1.71 | 0.705 |
| Diabetes mellitus | 1.02 | 0.45 | 2.29 | 0.971 | 0.87 | 0.42 | 1.83 | 0.717 |
| Ischemic stroke in past medical history | 2.13 | 0.64 | 7.06 | 0.214 | 1.77 | 0.59 | 5.28 | 0.309 |
| Acute ischemic stroke | 1.40 | 0.66 | 2.98 | 0.384 | 1.56 | 0.78 | 3.09 | 0.205 |
| LEOSS stage of disease: complicated^b^ | 1.34 | 0.38 | 4.77 | 0.651 | 0.81 | 0.28 | 2.34 | 0.697 |
| LEOSS stage of disease: critical^b^ | 1.72 | 0.48 | 6.14 | 0.399 | 1.39 | 0.49 | 3.94 | 0.533 |
| ECMO | 3.22 | 1.48 | 7.05 | 0.003 | 3.80 | 1.87 | 7.73 | <0.001 |
| Platelet count < 120,000 G/l^c^ | 0.59 | 0.18 | 1.98 | 0.393 | 0.64 | 0.21 | 1.93 | 0.428 |
| Platelet count 120,000-450,000 G/l^c^ | 0.65 | 0.16 | 2.63 | 0.548 | 0.72 | 0.20 | 2.58 | 0.612 |
| aPTT 25-39s^d^ | 1.90 | 0.40 | 9.01 | 0.416 | 1.72 | 0.47 | 6.35 | 0.411 |
| aPTT 40-54s^d^ | 1.69 | 0.30 | 9.57 | 0.551 | 1.56 | 0.36 | 6.68 | 0.547 |
| aPTT 55-69s^d^ | 8.28 | 1.55 | 44.35 | 0.014 | 5.56 | 1.31 | 23.62 | 0.020 |
| aPTT > 70s^d^ | 7.72 | 1.35 | 44.19 | 0.022 | 5.54 | 1.22 | 25.17 | 0.027 |
| INR 1.25-1.99^e^ | 1.21 | 0.54 | 2.72 | 0.635 | 1.01 | 0.47 | 2.15 | 0.982 |
| INR > 2^e^ | 0.65 | 0.10 | 4.23 | 0.647 | 0.92 | 0.17 | 4.91 | 0.921 |

**C.**

|  |  | | | |
| --- | --- | --- | --- | --- |
|  | **CIP/CIM** | | | |
| **Parameter** | **Odds-ratio** | **2.5% CI** | **97.5% CI** | **p** |
| (Intercept) | 0.06 | 0.01 | 0.62 | 0.018 |
| Age (years) ^a^ | 0.99 | 0.96 | 1.02 | 0.422 |
| Female sex | 0.91 | 0.44 | 1.91 | 0.805 |
| Time from ICU admission to first neurologic symptom (days) ^a^ | 1.05 | 1.03 | 1.08 | <0.001 |
| Diabetes mellitus | 1.36 | 0.64 | 2.89 | 0.426 |
| ARDS | 4.82 | 1.44 | 16.09 | 0.011 |
| Sepsis | 1.14 | 0.55 | 2.35 | 0.728 |
| Vasopressors | 1.31 | 0.31 | 5.53 | 0.713 |
| IL6 >= 80pg/ml^f^ | 0.70 | 0.32 | 1.53 | 0.364 |
| SAPS Score ≥ 50.5 | 0.39 | 0.10 | 1.48 | 0.160 |
| Acute liver failure | 0.87 | 0.32 | 2.37 | 0.785 |
| Acute kidney failure | 0.80 | 0.38 | 1.68 | 0.561 |
| Muscle relaxant drug | 0.90 | 0.44 | 1.82 | 0.760 |

**D.**

|  |  | | | |
| --- | --- | --- | --- | --- |
|  | **Delirium** | | | |
| **Parameter** | **Odds-ratio** | **2.5% CI** | **97.5% CI** | **p** |
| (Intercept) | 0.13 | 0.02 | 0.67 | 0.015 |
| Age (years) | 1.03 | 1.01 | 1.06 | 0.006 |
| Female sex | 0.40 | 0.21 | 0.74 | 0.004 |
| Alcohol consumption | 1.79 | 0.54 | 5.93 | 0.332 |
| Dementia in past medical history | 2.19 | 0.54 | 8.98 | 0.274 |
| Acute kidney failure | 1.32 | 0.75 | 2.32 | 0.336 |
| Acute Ischemic stroke | 0.06 | 0.03 | 0.15 | <0.001 |
| Acute ICH | 0.19 | 0.07 | 0.53 | 0.002 |
| Time from ICU admission to first neurologic symptom (days) | 0.98 | 0.96 | 1.00 | 0.031 |
| LEOSS stage of disease: complicated | 1.13 | 0.48 | 2.68 | 0.783 |
| LEOSS stage of disease: critical | 0.87 | 0.37 | 2.01 | 0.740 |
| IL6 > 80pg/ml | 0.99 | 0.54 | 1.84 | 0.986 |

Table 6a, b, c and d:

Abbreviations: ECMO: Extracorporeal membrane oxygenation, aPTT: activated partial thromboplastin time, INR: international normalized ratio, IL6: Interleukin 6

a Age/Duration/mRS were used as continuous covariates, odds ratios are given per point on the respective scale. To get, for example, the estimated odds ratio of 10 years of age, calculate 1.02^(10).

b Compared to LEOSS Stage uncomplicated

c Compared to patients with a platelet count of more than 450000/l

d Compared to patients with an aPPT of less than 25

e Compared to patients with an INR of less than 1.25

f Compared to patients with an IL6 of less than 80

**Supplement Table 7: Factors influencing the decision to therapy limitation**

|  | **Multivariable Full Model** | | | |
| --- | --- | --- | --- | --- |
| **Parameter** | **Odds-ratio** | **2.5% CI** | **97.5% CI** | **p** |
| (Intercept) | 0.00 | 0.00 | 0.02 | <0.001 |
| Cerebrovascular disorder | 3.59 | 1.80 | 7.14 | <0.001 |
| PNS disorder | 0.35 | 0.13 | 0.99 | 0.047 |
| Encephalopathy | 1.26 | 0.66 | 2.39 | 0.478 |
| Inflammatory CNS disorder | 1.84 | 0.16 | 21.08 | 0.625 |
| Epileptic Disorder | 0.87 | 0.35 | 2.16 | 0.771 |
| Age | 1.06 | 1.02 | 1.09 | <0.001 |
| Sex (w) | 0.96 | 0.53 | 1.74 | 0.890 |
| Pre-existing neurological diseases | 0.97 | 0.48 | 1.95 | 0.923 |
| Pre-existing cardiovascular diseases | 1.20 | 0.59 | 2.45 | 0.615 |
| Vasopressors | 2.46 | 0.78 | 7.76 | 0.124 |
| Invasive ventilation | 0.69 | 0.22 | 2.13 | 0.514 |
| ARDS | 1.05 | 0.47 | 2.35 | 0.913 |
| Acute kidney failure | 0.98 | 0.50 | 1.94 | 0.952 |
| Acute liver failure | 1.85 | 0.80 | 4.25 | 0.147 |
| Sepsis | 1.91 | 0.95 | 3.81 | 0.068 |
| ECMO | 0.96 | 0.42 | 2.16 | 0.912 |
| pmRS | 1.12 | 0.90 | 1.39 | 0.322 |
| LEOSS stage of disease: complicated | 1.02 | 0.41 | 2.57 | 0.963 |
| LEOSS stage of disease: critical | 1.10 | 0.41 | 2.92 | 0.850 |
| Anticoagulant drugs on admission | 1.13 | 0.53 | 2.41 | 0.744 |
| Time from COVID diagnosis to first neurologic deficit (days) | 0.98 | 0.96 | 1.00 | 0.112 |

ECMO: Extracorporeal membrane oxygenation, ARDS: Acute respiratory distress Syndrome
